# The characterization of actions at the superordinate, basic and subordinate level

**DOI:** 10.1007/s00426-021-01624-0

**Published:** 2021-12-14

**Authors:** Tonghe Zhuang, Angelika Lingnau

**Affiliations:** Chair of Cognitive Neuroscience, Faculty of Human Sciences, Institute of Psychology, University of Regensburg, Universitätsstrasse 31, 93053 Regensburg, Germany

## Abstract

Objects can be categorized at different levels of abstraction, ranging from the superordinate (e.g., fruit) and the basic (e.g., apple) to the subordinate level (e.g., golden delicious). The basic level is assumed to play a key role in categorization, e.g., in terms of the number of features used to describe these actions and the speed of processing. To which degree do these principles also apply to the categorization of observed actions? To address this question, we first selected a range of actions at the superordinate (e.g., locomotion), basic (e.g., to swim) and subordinate level (e.g., to swim breaststroke), using verbal material (Experiments 1–3). Experiments 4–6 aimed to determine the characteristics of these actions across the three taxonomic levels. Using a feature listing paradigm (Experiment 4), we determined the number of features that were provided by at least six out of twenty participants (common features), separately for the three different levels. In addition, we examined the number of shared (i.e., provided for more than one category) and distinct (i.e., provided for one category only) features. Participants produced the highest number of common features for actions at the basic level. Actions at the subordinate level shared more features with other actions at the same level than those at the superordinate level. Actions at the superordinate and basic level were described with more distinct features compared to those provided at the subordinate level. Using an auditory priming paradigm (Experiment 5), we observed that participants responded faster to action images preceded by a matching auditory cue corresponding to the basic and subordinate level, but not for superordinate level cues, suggesting that the basic level is the most abstract level at which verbal cues facilitate the processing of an upcoming action. Using a category verification task (Experiment 6), we found that participants were faster and more accurate to verify action categories (depicted as images) at the basic and subordinate level in comparison to the superordinate level. Together, in line with the object categorization literature, our results suggest that information about action categories is maximized at the basic level.

**Supplementary Information:**

The online version contains supplementary material available at 10.1007/s00426-021-01624-0.

## Introduction

Categorization is a cognitively economical way for humans to get to know and refer to the world (Rosch et al., 1976). That is, we tend to divide objects into different groups (also called categories), such as animals, tools, fruits, and vehicles. Categorization distinguishes one category from others, and at the same time reduces differences among objects falling into the same category, thus supporting effective recognition. At the same time, objects can be classified into different levels (superordinate, basic, and subordinate) based on the degree of abstraction (Rosch et al., 1976). For example, depending on our knowledge and the situation, we might label the food in front of us as a ‘fruit’ (superordinate level), an ‘apple’ (basic level), or a ‘golden delicious’ (subordinate level). In other words, objects have been proposed to be organized according to categories (which we refer to as ‘horizontal organization’) as well as to a hierarchy (referred to as ‘vertical organization’ in the remainder of this article).

Rosch et al. (1976) suggested that the principles of categorization are cognitive economy and cue validity. Cognitive economy refers to the balance between the ability to categorize objects at a high level of precision (e.g., using a granny smith rather than a golden delicious for your favorite apple pie), while at the same time building in enough flexibility to limit the capability of this system to the distinctions required by a given task or circumstances (e.g., by knowing that an apple can have a range of sizes and different colors from red over yellow to green, while the shape is always round). Cue validity refers to the validity that a given cue (e.g., ‘round’, ‘green’, ‘slightly sour’) serves as a predictor of a certain category (e.g., ‘granny smith’). Cue validity increases the association between the cue and the category and decreases the association between the cue and other categories.

In a series of experiments, Rosch et al. (1976) investigated the informational capacity of objects at the superordinate, basic and subordinate level. Using a feature listing paradigm, participants were instructed to write down as many features as they could think of for objects at each level. Features that were provided by at least six out of 20 participants were considered common features, i.e., features that reflect the cue validity of an object. The authors observed that participants provided more common features for objects at the basic level in comparison to the superordinate level. Participants provided the smallest number of common features for objects at the superordinate level. Assuming that lower taxonomic levels include all the features listed at the higher level, Rosch et al. (1976) determined the number of features added at the basic level (in comparison to the superordinate level) and the number of features added at the subordinate level (in comparison to the basic level). They observed that a smaller number of features was added at the subordinate level than at the basic level and concluded that the basic level is most inclusive in terms of the attributes objects at that level have in common. Moreover, Rosch (1978) argued that objects at the subordinate and superordinate level have a lower cue validity in comparison to the basic level, because (a) many common features were shared with other objects from other categories at the subordinate level, and (b) objects at the superordinate level were described with a large proportion of distinctive features.

In a separate experiment, Rosch et al. (1976) instructed participants to list motor (body and muscle) movements associated with specific objects. They found that participants listed fewer motor movements for objects at the superordinate level in comparison to objects at the basic and subordinate level. By contrast, participants listed a similar number of motor movements associated with objects at the basic and subordinate level. According to the authors, these results illustrated that the basic level was the most inclusive level at which many motor movements are associated with objects.

Using picture-word-matching and priming paradigms, Rosch et al. (1976) found that object categories were recognized faster at the basic and subordinate level in comparison to the superordinate level. Using an object recognition task, the authors reported that participants verified object images fastest for object names at the basic level. Likewise, Rosch et al. (1976) reported that children learn to categorize objects at the basic level prior to objects at the superordinate or subordinate level. In sum, Rosch et al. (1976) concluded that objects at the basic level are associated with the highest cue validity.

More recent studies investigated the neural representation of objects at different hierarchical levels (Gauthier et al., 1997; Grill-Spector & Kanwisher, 2005; Grill-Spector et al., 2004; Iordan et al., 2015; Margalit et al., 2020). As an example, Gauthier et al. (1997) examined which brain areas are recruited during the processing of objects at the basic (e.g., bird) and the subordinate level (e.g., eagle) using a picture-word matching paradigm and a semantic judgement task. They observed a stronger recruitment of the fusiform gyrus, the inferior temporal gyrus and the occipital cortex during the judgement of objects at the subordinate level in comparison to judgements of objects at the basic level during both tasks. Likewise, using multi-voxel pattern analysis (MVPA), Iordan et al. (2015) reported representations of object categories at the subordinate in early visual cortex, whereas representations at the basic level were found both in early visual cortex and in object-selective regions. Representational similarity analysis (RSA, also a form of MVPA) revealed that the perirhinal cortex uniquely represents object categories at the superordinate level (animals, fruit, vegetables, tools, vehicles and musical instruments; Clarke & Tyler, 2014). Finally, different levels of the hierarchy have been argued to be represented at different spatial scales (e.g., Grill-Spector & Weiner, 2014; Margalit et al., 2020). Taken together, these studies demonstrate that the different hierarchical levels of objects proposed by Rosch et al. (1976) can also be distinguished at the neural level.

To which degree does the horizontal and vertical organization of objects described so far apply to the organization of actions? Note that this question is of theoretical interest both for the organization of actions we perform ourselves and actions we observe. Whereas there exist a number of interesting cross-links between these two lines of research that we will refer to, the current study focuses on the organization of observed actions. Similar to objects, observed actions have been suggested to be organized horizontally according to several superordinate categories, such as manipulation, locomotion and communication (Corbo & Orban, 2017; Tucciarelli et al., 2019; Wurm et al., 2017). Likewise, principles related (but not identical) to the vertical organization of objects can be found in the literature on the organization of actions. The importance of different levels of representations of actions has been explicitly spelled out in the Theory of Action Identification (Vallacher & Wegner, 1985; Wegner & Vallacher, 1986). This theory emphasizes the relationship between processes involved in comprehending and performing an action. Importantly, the theory proposes that actions can be identified at different levels, with lower levels related to the concrete implementation of an action, whereas higher levels provide a more abstract representation of an action reflecting the reasons and effects of the action. Likewise, Hamilton and Grafton (2006, 2008) and Grafton and Hamilton (2007) suggested three hierarchical levels of the motor system: the goal level (the purpose and outcome of action), the kinematic level (the shape and movement of hands and arms) and the muscle level (active patterns of the muscles). Using repetition suppression, they observed that the anterior intraparietal sulcus (aIPS) represents goals of observed actions (e.g., to grasp an object; Hamilton & Grafton, 2006). By contrast, kinematic aspects of the observed action have been reported to be represented in the lateral occipital cortex (LOC), superior parietal lobe, the fusiform cortex and the superior temporal sulcus (Hamilton & Grafton, 2006, 2008; for related studies, see also Grafton & Hamilton, 2007; Spunt et al., 2016). Using MVPA, Wurm and Lingnau (2015) distinguished between representations of observed actions (opening vs. closing an object) at a concrete level, referring to specific combinations of objects, kinematics and grip types, and an abstract level, which showed generalization across objects and kinematics. Their data revealed representations of actions at the concrete level in the lateral occipitotemporal cortex (LOTC), the inferior parietal lobe (IPL) and the ventral premotor cortex (PMv), whereas representations at the abstract level were restricted to the LOTC and the IPL. Finally, using MVPA, several studies revealed spatially distinct representations of executed actions at the goal level and the kinematic level (Gallivan et al., 2013; Kadmon Harpaz et al., 2014; Turella et al., 2020). Together, these studies are in line with the view that the representation of observed and executed actions follow a hierarchical organization, and that these can be distinguished at the neural level.

What the proposed hierarchies of objects and actions have in common is that the three proposed hierarchical levels differ with respect to their degree of abstractness. The main point in which they differ, however, is that the action hierarchies described above focused on the goal of the action and the different means by which these goals can be achieved. Given an action goal (such as eating a piece of cake), what are the underlying essential elements (such as cutting the cake, placing a piece of cake on a plate, picking up a fork) that are required to achieve it? By contrast, the taxonomy of objects proposed by Rosch et al. (1976) emphasizes the importance of object representations at the basic level, given that attributes provided for objects at this level have the highest cue validity, and that objects at this level are recognized faster than objects at the other two levels. Do the characteristics of objects at the superordinate, basic, and subordinate level reported by Rosch et al. (1976) also hold for the organization of observed actions?

Experiments 1–3 were conducted to create a set of actions (using verbal material) with a clear taxonomic structure. Since we aimed to use this stimulus set for future behavioral and neuroimaging studies, we wished to arrive at a final set of three to four superordinate categories and at least two basic and subordinate level actions within each of these superordinate categories. Moreover, since we anticipated that we would have to remove some of the actions for practical reasons (e.g., due to imbalances regarding ratings of abstractness or complexity), we started out with a broader range of actions than the final numbers we aimed to reach. Experiments 4–6 aimed to characterize the actions selected in Experiments 1–3 at the three taxonomic levels. Experiment 4 used a feature listing paradigm to determine the numbers of common, distinct and shared features across taxonomic levels. In Experiment 5 and 6, using visual material, we examined whether the three taxonomic levels differ in terms of priming and speed of recognition.

## Materials and methods

### Experiment 1 (stimulus selection, part I): rationale

In Experiment 1 we aimed to lay the foundation for the definition of a range of different actions at the three taxonomic levels. As a starting point, we selected action verbs corresponding to the basic level from Levin (1993), who suggested a hierarchical organization of English verb classes based on similarities of verb meaning and syntactic expressions. Note that the classes of verbs from Levin are based on linguistic criteria rather than explicit human judgment. Thus, to determine whether explicit ratings of semantic similarity reveal similar clusters as those proposed by Levin (1993), we instructed participants to provide ratings for pairs of action verbs in terms of the similarity of their meaning. To select suitable actions for our stimulus set from at least three superordinate categories, we carried out hierarchical cluster analysis.

### Experiment 1: methods

#### Participants

Twenty-one native German speakers (female: 12, age: 22.3 ± 4.2 years) took part in the experiment. All participants received written instructions about the experimental procedures and were reimbursed for their participation. Participants consented to participate in the study via button click. Procedures were approved by the local Ethics Committee at the University of Regensburg.

#### Stimuli

We selected german translations for 35 verbs from eight categories (communication, locomotion, ingesting, change of state, learning, grooming and body care, perception, creation and transformation). For the aim of future behavioral experiments (see, e.g., Experiments 5 and 6) for which we aimed to use images of actions, we focused on action verbs that we considered suitable to be depicted as images. The list of verbs in their infinitive form, together with their German translations and the corresponding categories is provided in Table S1.

#### Procedure

During the experiment, action phrases consisting of verbs in their infinitive form were presented in pairs on the screen. Participants were instructed to rate the semantic similarity of these pairs of action phrases on a scale from 1 (very dissimilar) to 7 (very similar). The rating procedure was carried out using an online survey (SoSciSurvey) and took approximately 20 min per participant. Details of the instruction are provided in the Supplementary Material (Experiment 1 section).

#### Data analysis

Data analysis was carried out in MATLAB. To examine taxonomies of verbs, we conducted hierarchical cluster analysis on the provided semantic similarity ratings and visualized the results as a dendrogram. To determine the optimal number of clusters, we computed the silhouette index (Rousseeuw, 1987). The silhouette index provides an estimate of the average distance between clusters—the higher the index, the better the items cluster.

### Experiment 1: results

**Fig. 1:**
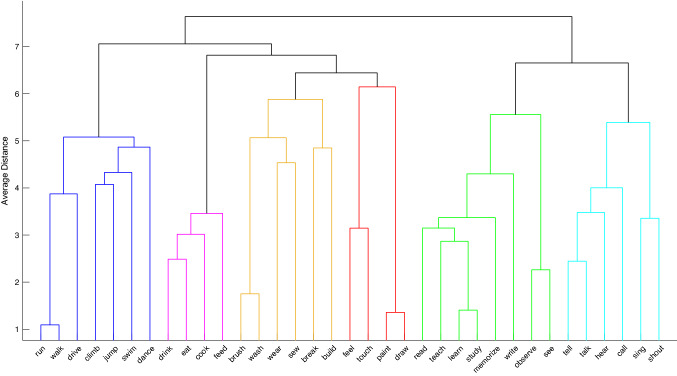
Dendrogram illustrating the results of the hierarchical clustering analysis. Actions belonging to the same cluster are highlighted in the same color. Blue: locomotion, purple: ingestion, yellow: object manipulation, red: sensation/leisure-related actions, green: learning/studying, turquoise: communication

The silhouette analysis indicated that the optimal number of clusters was six (Fig. S1). The corresponding clusters are shown in Fig. 1 and consist of actions related to locomotion, ingestion, object manipulation, sensation/leisure-related actions, learning/studying, and communication (see also Tucciarelli et al., 2019). The results showed similarities with the classification proposed by Levin (1993), but we also obtained some differences. For example, we obtained a cluster consisting of ‘to see’, ‘to observe’, ‘to write’ and ‘to read’ that was distinct from another cluster that contained the verbs ‘to feel’, ‘to touch’, ‘to paint’ and ‘to draw’. By contrast, in Levin’s classification, ‘to see’ and ‘to observe’ were clustered together with ‘to feel’ and ‘to touch’. These differences are not unexpected, given that Levin’s classification was based on associations between syntactic properties of English verbs and their meanings, whereas the current study used explicit ratings of semantic similarities of verbs provided in German. We wish to point out that while it is interesting to notice these differences, a systematic comparison between the classification proposed by Levin and the results of the explicit ratings of semantic similarity was outside the scope of the current study.

### Experiment 2 (stimulus selection, part II): rationale

From the results of Experiment 1, we obtained six clusters (which we considered to correspond to actions at the superordinate level) and their members (which we considered to correspond to actions at the basic level). The goal of Experiment 2 was to select and establish the superordinate, basic, and subordinate level structure of actions with an independent set of participants using a taxonomic depth task (see also Rifkin, 1985). To this aim, participants were instructed to write down labels for members of basic level actions (corresponding to subordinate level actions), and to provide labels (corresponding to superordinate categories) for the clusters revealed in Experiment 1.

### Experiment 2: methods

#### Participants

Twenty native German speakers (female: 17, age: 21.5 ± 1.5 years) participated in Experiment 2. All participants were informed about the study procedures and received payment for participating. Participants consented to participate in the study via button click. Procedures were approved by the local Ethics Committee at the University of Regensburg.

#### Stimuli

We used a subset of the German action verbs used in Experiment 1. First, we excluded action verbs that might be ambiguous when depicted as a picture rather than a word (e.g., to learn—lernen, to teach—unterrichten, to read—lesen). Moreover, we excluded action verbs for which their subordinate actions could have been difficult to identify as a picture (e.g., to memorize—merken). Based on these criteria, we excluded two superordinate categories (sensation/leisure-related actions: to feel, to touch, to paint, to draw; learning/studying: to read, to teach, to learn, to study, to memorize, to write, to observe, to see). Following the same criteria, we kept two to three basic level actions for each of the remaining superordinate categories for the taxonomic depth task (locomotion: to walk, to drive, to swim; ingestion: to drink, to eat, to cook; object manipulation: to brush, to wash; communication: to tell, to talk, to hear).

#### Procedure

The experiment consisted of a taxonomic depth task similar to the one used by Rifkin (1985). It was carried out with an online questionnaire (http://www.soscisurvey.com) and consisted of two parts. In the first part, participants were asked to provide names of actions at the subordinate level. In each trial, one of the action verbs at the basic level (e.g., to drive/fahren) was displayed on the top of the screen. Participants were instructed to write down at least two subordinate names within 30 s, such as ‘to ride a bike’ (Fahrrad fahren) or ‘to drive a car’ (Auto fahren). The order of the action verbs was randomized.

In the second part, participants were asked to provide superordinate names for clusters of actions. During each trial, participants were provided with those two to three verbs belonging to individual clusters obtained from Experiment 1 (e.g., to walk, to drive, to swim), and were instructed to type the superordinate name these verbs belong to (e.g., locomotion) within 30 s. The order in which clusters were presented to participants was randomized. The whole experiment took approximately 10 min. Instructions for both parts are provided in the Supplementary Material.

#### Data analysis

After obtaining names of actions at the subordinate (first part) and superordinate (second part) level, two German native speakers combined similar answers into one answer and then labelled it. For instance, ‘Bewegung’ and ‘Fortbewegen’ was combined together and labelled ‘Bewegung’ (locomotion). For each cluster at the superordinate level, we chose one final label based on the most frequently used labels. For the subordinate level, we kept/chose the two or three action verbs with the highest frequencies.

### Experiment 2: results

The results of Experiment 2 are summarized in Table 1, showing labels obtained for superordinate (left column) and subordinate level actions (right column) next to the basic level actions (middle column) that served as the input to the taxonomic depth task. Note that due to the nature in which we generated the subordinate level actions, we did not control for the use of object versus non-object-directed actions.

**Table 1:** Labels for action categories at the superordinate and subordinate level resulting from the taxonomic depth task (Experiment 2)

Superordinate level	Basic level	Subordinate level
Locomotion (sich fortbewegen)	To go (gehen)	To walk (spazieren gehen)
		To hike (wandern)
		To walk a dog (Gassi gehen)
	To drive (fahren)	To drive a car (Auto fahren)
		To ride a bike (Fahrrad fahren)
		To take a bus (Bus fahren)
	To swim (schwimmen)	To swim front crawl (Kraulschwimmen)
		To swim breaststroke (Brustschwimmen)
Ingestion (Nahrung aufnehmen)	To drink (trinken)	To drink water (Wasser trinken)
		To drink beer (Bier trinken)
		To drink coffee (Kaffee trinken)
	To eat (essen)	To eat an apple (einen Apfel essen)
		To eat cake (Kuchen essen)
	To cook (kochen)	To cook noodles (Nudeln kochen)
		To cook soup (Suppe kochen)
Cleaning (sauber machen)	To brush (putzen)	To clean windows (Fenster putzen)
		To brush teeth (Zähne putzen)
		To clean the bathroom (Bad putzen)
	To wash (waschen)	To wash clothes (Wäsche waschen)
		To do the dishes (Geschirr abwaschen)
		To clean the face (Gesicht waschen)
Communication (Kommunizieren)	To talk (sich unterhalten)	To talk to friends (sich mit Freunden unterhalten)
		To talk on the phone (sich am Telefon unterhalten)
	To listen (Hören)	To listen to someone (jemandem zuhören)
		To listen to the radio (Radio hören)
	To tell (erzählen)	To tell a joke (einen Witz erzählen)
		To tell a story (eine Geschichte erzählen)

Basic level actions were selected on the basis of Experiment 1

### Experiment 3 (stimulus selection, part III): rationale

The purpose of Experiment 3 was twofold. First, to further validate the relationship between actions at the subordinate and the superordinate level resulting from Experiment 2, we instructed a new set of participants to explicitly rate the relationship between actions at these two levels. Second, for the purpose of future behavioral and neuroimaging studies, we aimed to balance actions at the subordinate level with respect to their degree of abstraction and complexity. The reason for this is that both complexity and the level of abstraction are known to have an impact on behavioral measures as well as on the corresponding neuronal signatures (e.g., Breedin et al., 1998; Gennari & Poeppel, 2003; Moseley & Pulvermüller, 2014; Schwanenflugel, 1991; Wang et al., 2010). To this aim, we asked participants to rate the degree of abstraction and complexity of each action at the subordinate level. We used these ratings to identify and remove ‘unsuitable actions’ that we treated as outliers, since they differed from the other actions at the subordinate level in terms of the level of abstractness and the level of complexity, and since the judged relationship between the subordinate and the superordinate level for these actions was either too low within a category or too high between categories.

### Experiment 3: methods

#### Participants

Twenty-five native German speakers (female: 16, age: 22.0 ± 6.0 years) were recruited to take part in this experiment. All participants were informed about the study procedures and received payment for participating. Participants consented to participate in the study via button click. Procedures were approved by the local Ethics Committee at the University of Regensburg.

#### Stimuli

We used the german action category labels at the subordinate and superordinate level resulting from Experiment 2 (see Table 1).

#### Procedure

Both parts of the experiment were performed using an online platform (http://www.soscisurvey.com). For the rating of the relationship of actions at the superordinate and subordinate level, separately for each superordinate category, the superordinate category label of an action (e.g., locomotion) was shown at the top of the screen and all 27 subordinate level actions (e.g., to walk a dog, to hike, …, to tell a story; see Table 1) were shown underneath. Participants were instructed to assess the relation of each action at the subordinate level with the action at the superordinate level on a scale from 1 (weak relationship) to 7 (strong relationship).

For the rating of abstraction and complexity, participants were instructed to rate the degree of abstraction and the degree of complexity on a scale from 1 to 7 (1: very concrete, 7: very abstract; 1: very simple, 7: very complex). Further details about the instruction are provided in the Supplementary Material.

#### Data analysis

First, to remove outliers within a category for ratings of relatedness, we used the median absolute deviation (MAD; Leys et al., 2013). Second, after removing outliers we used the Mann–Whitney *U* test to compare ratings of relatedness of actions at the superordinate and superordinate level between within-category (e.g., locomotion—to walk a dog) and between-category (e.g., locomotion—to eat an apple) pairs of actions. Third, we removed outliers based on ratings of abstraction and complexity using ± 1.5 MAD.

### Experiment 3: results

The ratings of the relationship between actions at the superordinate and the subordinate level within and across categories are shown in Table 2. Based on the MAD, we removed the subordinate level action ‘to listen to the radio’, because its relation to the superordinate category ‘communication’ was considered too low (rating = 2.96, median = 5.82, lower bound = 4.12).

**Table 2:**
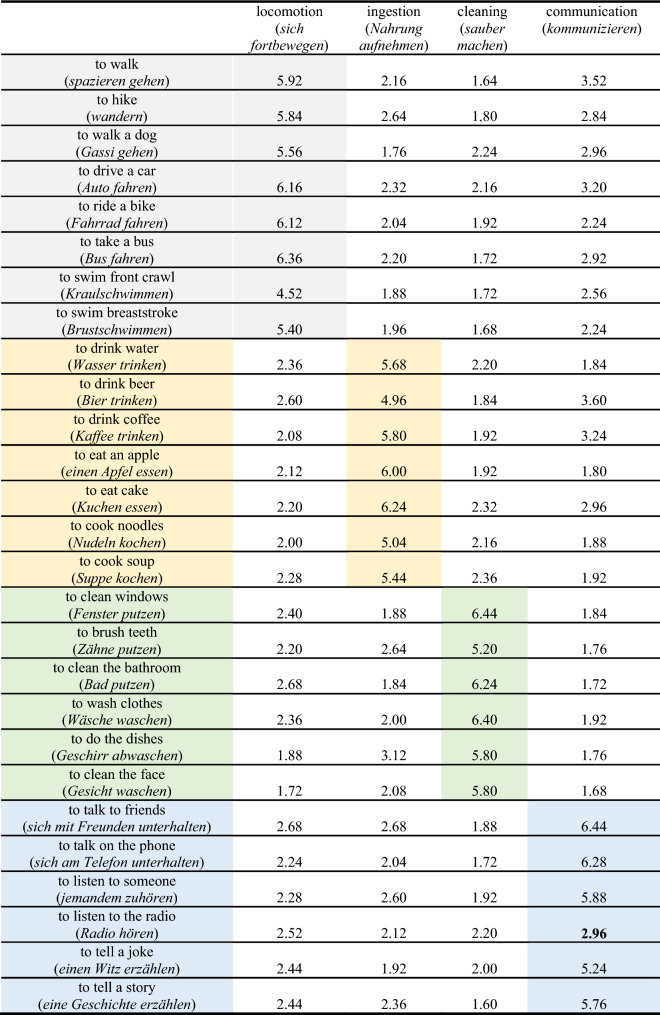
Relationship between actions at the superordinate (columns) and subordinate (rows) level within (highlighted in grey, yellow, green, and blue) and across categories (1: very weak relationship; 7: very strong relationship)

Note: Actions that were rated as outliers using MAD analysis (Leys et al., 2013) are marked in bold and were removed from further analyses

As expected, the median rating for the relationship between actions at the superordinate and the subordinate level was higher for subordinate level actions belonging to the same superordinate category (e.g., ingestion—to drink water; median = 5.80) than for subordinate level actions belonging to a different superordinate category (e.g., ingestion—to hike; median = 2.16). This observation was supported by the Mann–Whitney *U* test [*U*_(*N* within category = 27, *N* between categories = 81)_ = 6.00, *z* = − 7.72, *p* < 0.001, (*N*: sample size)].

Ratings of abstraction and complexity are provided in Table 3. The actions ‘to tell a joke’ and ‘to tell a story’ were removed, since their ratings of abstraction were detected as outliers by the MAD. Finally, we excluded the category ‘communication’ from further experiments, because only one action at the basic level was left after these actions were excluded.

**Table 3:**
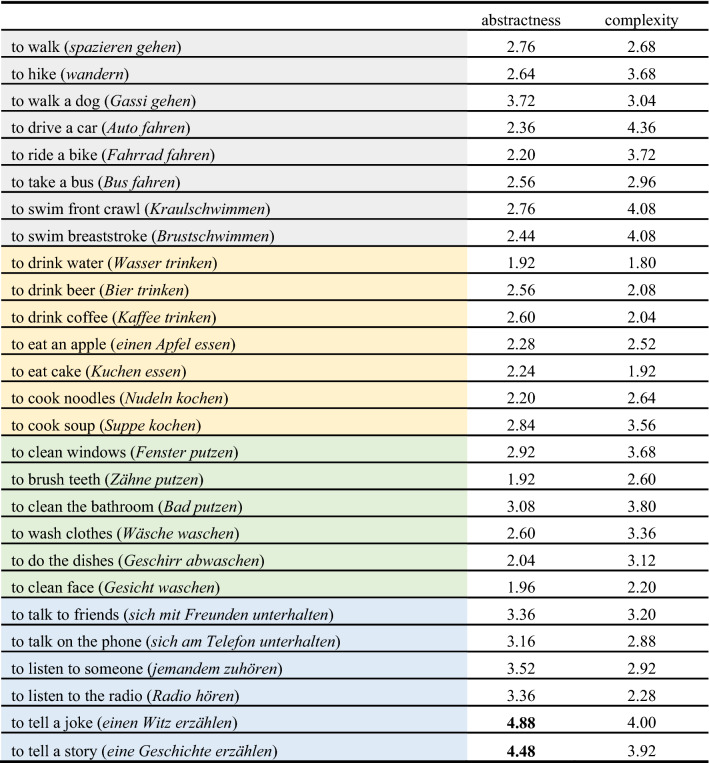
Ratings of abstraction (1: very concrete, 7: very abstract) and complexity (1: very simple, 7: very complex)

Note: Mean ratings of abstraction and complexity for actions provided at the subordinate level belonging to one of four different superordinate levels (grey: ‘locomotion’; yellow: ‘ingestion’; green: ‘cleaning’ and blue: ‘communication’). Actions that were determined as outliers using MAD analysis (Leys et al., 2013) are marked in bold and were removed

### Experiment 4 (feature listing): rationale

The purpose of Experiment 4 was to examine the characteristics of actions at the three different taxonomic levels. To this aim, separately for each action at the superordinate, basic and subordinate level, participants were instructed to list features. As an example, the features of the action ‘to write’ could be ‘hand’, ‘fingers’, ‘paper,’ and ‘type’. Following Rosch et al. (1976), we determined the number of common features (see “Data analysis” for details). Moreover, we further divided common features into shared and distinct features (see also Rosch, 1978). Following Rosch et al. (1976), shared features are the common features that are shared with other categories, whereas distinct features refer to those features that belong to one category only. We hypothesized that participants provide more common features for actions at the basic level in comparison to the other two levels. Moreover, we hypothesized that participants provide the fewest common features for actions at the superordinate level, and that actions at the subordinate level share most features with actions from other categories at the same level.

### Experiment 4: methods

#### Participants

To prevent that providing features at one level influences the ratings of features provided at another level, three separate groups of participants took part in the experiment for the listing of features at the superordinate, basic and subordinate level. Each group consisted of *N* = 20 native German speakers (group 1: 17 females, age: 22.9 ± 3.2 years; group 2: 15 females, age: 28.1 ± 10.5 years; group 3: 12 females, age: 21.1 ± 4.5 years). All participants were informed about the study procedures and were reimbursed for participating in the study. Participants consented to participate in the study via button click. Procedures were approved by the local Ethics Committee at the University of Regensburg.

#### Stimuli

As in Experiments 1–3, all action phrases were provided in German. Based on the results of Experiment 3, we excluded the german translations for ‘to listen to the radio’, ‘to tell a story’ and ‘to tell a joke’. Due to the small number of remaining actions, we decided to remove all actions corresponding to the cluster ‘communication’. Thus, we selected the superordinate level categories ‘locomotion’, ‘ingestion’ and ‘cleaning’ as well as their members at the subordinate level. Note that we did not control for co-occurences between different subordinate actions on the basis of the nouns included in the action phrases (e.g., to drink beer—Bier trinken; to clean the face—das Gesicht waschen).

To balance the number of actions within each category for the final set of actions, we chose two basic action categories for each action at the superordinate level and two subordinate action categories for each action at the basic level (see Table 4). Finally, to verify whether action phrases at the three taxonomic levels differ with respect to their degree of abstraction, we asked a separate group of *N* = 20 participants to rate the degree of abstraction (1: very concrete; 7: very abstract), separately for each of the action phrases provided in Table 4 (see Supplementary Material, Experiment 4 section, for details). As expected, ratings for abstraction were highest for the superordinate level (mean rating: 5.10), intermediate for the basic level (mean rating: 4.03), and lowest for the subordinate level (mean rating: 1.45). These observations are supported by the corresponding statistics (*H*_(2)_ = 288.75, *p* < 0.0001, ${\eta }^{2}$ = 0.69).

**Table 4:** Actions at the superordinate, basic and subordinate level used in Experiments 4–6

Superordinate level	Basic level	Subordinate level	
Locomotion (sich fortbewegen)	To drive (fahren)	To drive a car (Auto fahren)	
		To take a bus (Bus fahren)	
	To swim (schwimmen)	To swim front crawl (Kraulschwimmen)	
		To swim breaststroke (Brustschwimmen)	
Ingestion (Nahrung aufnehmen)	To drink (trinken)	To drink water (Wasser trinken)	
		To drink beer (Bier trinken)	
	To eat (essen)	To eat an apple (einen Apfel essen)	
		To eat cake (Kuchen essen)	
Cleaning (sauber machen)	To brush (putzen)	To clean the windows (Fenster putzen)	
		To brush teeth (Zähne putzen)	
	To wash (waschen)	To do the dishes (Geschirr abwaschen)	
		To clean the face (Gesicht waschen)	

#### Procedure

Following the original procedures by Rosch et al. (1976), we carried out a free feature listing paradigm implemented as an online survey (http://www.soscisurvey.com) to investigate characteristics of actions at the taxonomic different levels. Three different groups of participants were presented with phrases describing actions at either the superordinate, basic or subordinate level. During each trial, a german action phrase consisting of a verb in its infinitive form (e.g., to drink water—Wasser trinken) was shown at the top of the page. Participants were instructed to write down as many features as possible related to that action within 2 min. The features could be related to body parts involved in the action, the target of the action, the type of movements involved, specific postures, the duration, the use of force, the pace etc. For example, features for ‘to drink water’ could be ‘glass’, ‘water’, ‘mouth’, ‘hand’, ‘bend’, etc. Features of ‘to drink’ could be ‘hand movement’, ‘water’, ‘beer’, ‘swallow’ and so on. Features of ‘ingestion’ could be ‘food’, ‘swallow’, ‘mouth’, ‘pour’, ‘hand’, etc. Details regarding the instructions are provided in the Supplementary Material.

#### Data analysis

To identify reliable features of each action per level, four German speakers judged these features. Features referring to the same meaning (e.g., ‘rag’ and ‘cleaning rag’) were merged (‘rag’). Answers that contained several features (e.g., ‘rotating arm’) were separated into individual features (e.g., ‘to rotate’, ‘arm’). By contrast, if only the combination of two features (e.g., ‘frische Luft’—‘fresh air’) but not the two individual features (‘frische’—‘fresh’, ‘Luft’—‘air’) referred to the action, we counted the combined feature rather than the individual features. The four judges were provided with a detailed coding scheme explaining these rules, together with concrete examples. The judge-amended tallies of each feature for each action were determined for further statistical comparisons.

To investigate the characteristics of actions at each taxonomic level, we computed the number of common features (the number of features provided by at least six out of 20 participants), separately for each of the three levels. Next, we separated common features into shared and distinct features at the three different levels to determine the distinctions of features. On the basis of the common features, distinct features were defined as features that were provided for one category only whereas shared features were determined as those that were provided for more than one category. Moreover, to examine distinctions between feature types, we distinguished common features into movement features, body-part features and object features.

To examine differences between the three hierarchical levels in terms of the mean number of common, distinct and shared features, we used the Kruskal–Wallis *H* test, which is a rank-based nonparametric test for the comparison of two or more independent samples with equal or unequal sample sizes. Next, we used Dunn’s post hoc tests and corrected for multiple tests using Bonferroni correction. All statistical analyses were implemented in SPSS (https://www.ibm.com/analytics/spss-statistics-software). Effect sizes for the Kruskal–Wallis *H* test are reported as ${\eta }^{2}$.

### Experiment 4: results

**Fig. 2:**
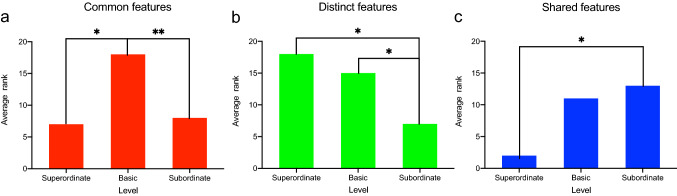
Mean number of common, distinct and shared features of actions at the superordinate, basic and subordinate level. **a** Actions at the basic level were described with more common features than actions at the other two levels. **b** Actions at the superordinate and basic level were described with more distinct features than actions at the subordinate level. **c** Actions at the subordinate level were described with more shared features than actions at the superordinate level

As can be seen in Fig. 2, actions at the basic level were described with more common features in comparison to the other two levels (panel A). At the same time, actions were described with more distinct features at the superordinate and the basic level in comparison to the subordinate level (panel B). Actions at the subordinate level were described with more shared features than actions at the superordinate level (panel C). These observations are supported by the corresponding statistics (see following section).

To compare different types of features between the three taxonomic levels, we used the Kruskal–Wallis *H* test. The mean rank of common features (Fig. 2a) differed significantly between the three different levels (*H*_(2)_ = 11.68, *p* = 0.003, ${\eta }^{2}$ = 0.58; see Table 5 for details). Furthermore, the mean rank of common features of actions was 7.00 (numbers of actions (*n*) = 3) at the superordinate level, 18.17 (*n* = 6) at the basic level, and 8.42 (*n* = 12) at the subordinate level. Dunn’s post-hoc tests indicated that participants provided more common features at the basic level in comparison to the superordinate (basic vs. superordinate level: *p* = 0.03) and the subordinate level (basic vs. subordinate level: *p* = 0.004). However, we obtained no significant difference of common features between actions at the superordinate and subordinate level (*p* > 0.99).

**Table 5:** Results of the Kruskal–Wallis *H* test for common, distinct and shared features (upper part), and for common features, separately for movement, body part and object features (lower part)

	Test statistic	Total number	df	Sig.	${\eta }^{2}$
Common features	11.68	21	2	0.003***	0.58
Distinct features	11.81	21	2	0.003***	0.59
Shared features	7.77	21	2	0.021*	0.39
Common features					
Movement features	7.67	21	2	0.022*	0.38
Body part features	1.03	21	2	0.317	0.05
Object features	4.39	21	2	0.314	0.22

Note: *p < 0.05; **p < 0.01; ***p < 0.005; ****p < 0.001

The mean rank of distinct features (Fig. 2b) differed significantly between the three taxonomic levels (*H*_(2)_ = 11.81, *p* = 0.003, ${\eta }^{2}$ = 0.59). The mean rank of distinct features of actions provided by participants was 17.67 (*n* = 3) at the superordinate level, 15.58 (*n* = 6) at the basic level, and 7.04 (*n* = 12) at the subordinate level. Dunn’s post-hoc test revealed that participants described actions at the superordinate and basic level with more distinct features than actions at the subordinate level (superordinate vs. subordinate level: *p* = 0.02; basic vs. subordinate level: *p* = 0.02). However, there was no significant difference in distinct features between the superordinate and basic level (*p* > 0.99).

The mean rank of shared features (Fig. 2c) differed significantly between the three levels (*H*_(2)_ = 7.77, *p* = 0.02, ${\eta }^{2}$ = 0.39). The mean rank of shared features was 2.00 (*n* = 3) at the superordinate level, 11.50 (*n* = 6) at the basic level, and 13.00 (*n* = 12) at the subordinate level. Dunn’s post-hoc tests showed that participants provided more common features that were shared with other categories at the subordinate level compared to actions at the superordinate levels (*p* = 0.02). The number of shared features did not differ between actions at the basic and subordinate level (*p* > 0.99), and between actions at the basic and superordinate level (*p* = 0.09).

To explore differences between different types of features, we computed the number of common features separately for movement features, body-part features, and object features. The Kruskal–Wallis *H* test showed that the three levels differed in terms of the number of movement features (*H*_(2)_ = 7.67, *p* = 0.02, ${\eta }$ = 0.38, Table 5). The mean rank of movement features at the different levels were 5.00 (*n* = 3) at the superordinate level, 16.17 (*n* = 6) at the basic level, and 9.92 (*n* = 12) at the subordinate level. Post hoc tests revealed that participants provided more movement-related features at the basic in comparison to the superordinate level (*p* = 0.03). The number of movement features did not differ between actions at the subordinate and superordinate level (*p* = 0.63), or between actions at the basic and subordinate level (*p* = 0.12). In contrast to movement features, the number of body-part features (*H*_(2)_ = 1.03, *p* = 0.317, ${\eta }^{2}$ = 0.05) and object features (*H*_(2)_ = 4.39, *p* = 0.314, ${\eta }^{2}$ = 0.22) was not modulated by the taxonomic level.

### Experiment 4: interim discussion

The results from Experiment 4 revealed that the types of features provided by participants differed between the taxonomic levels, with the largest number of common features obtained at the basic level, the largest number of distinct features at the superordinate level and the largest number of shared features at the subordinate level. Regarding the number of common features, Rosch et al., (1976, experiment 1) reported more common features at the basic in comparison to the superordinate level, in line with our results. Rosch et al. (1976) argued that this may illustrate the distinctiveness of objects at the superordinate level. The larger number of distinct features at the superordinate in comparison to the subordinate level obtained in the current study is compatible with this interpretation.

Rosch et al. (1976) furthermore reported that the number of features added at the subordinate level (in comparison to the basic level) was smaller than the number of features added at the basic level (in comparison to the superordinate level). They argued that less information was added at the subordinate level, because at this level, many features were shared with other categories, and concluded that objects at the subordinate level have lower cue validity in comparison to objects at the basic level (Rosch, 1978). Note that in the current study, participants provided fewer common features at the subordinate in comparison to the basic level. Whereas Rosch et al. (1976) did not report the statistics between the number of common features beetween the basic and the subordinate level, it is obvious from their Table 2 that the number of common features provided at the subordinate level was larger in comparison to the basic level. We are uncertain regarding the reasons for this discrepancy between the results of Rosch et al. (1976) and our results. That said, the larger number of shared features for actions at the subordinate in comparison to the superordinate level obtained in the current study suggests that actions at the subordinate level have lower cue validity in comparison to actions at the superordinate level.

In sum, the results of Experiment 4 reveal systematic differences regarding the types of features provided for actions at the three taxonomic levels that are broadly consistent with the results reported by Rosch et al. (1976). To examine whether these differences are reflected in terms of the speed of processing, we followed up these results with an auditory priming experiment (Experiment 5).

### Experiment 5 (priming): rationale

This experiment had two purposes. First, we aimed to examine at which taxonomic level actions are recognized first when depicted as pictures. Second, we aimed to determine whether participants are faster in processing the picture of an action if it is preceded by a matching (in comparison to a non-matching) action label (presented as an auditory cue), and if so, to which degree this priming effect is affected by the taxonomic level. To address these questions, we carried out a category verification task in which participants were presented with action labels (presented as auditory cues) at the subordinate, basic or superordinate level, followed by the image of an action. The task was to judge via button press whether the image corresponded to the action phrase. We hypothesized that participants verified actions at the basic level more rapidly than actions at the other two levels, and that participants responded faster and more accurately in matched in comparison to non-matched trials.

### Experiment 5: methods

#### Participants

Twenty-three native German speakers (female: 20; age: 21 ± 3 years) took part in this experiment via the online platform lab.js (https://labjs.readthedocs.io/en/latest/). Participants were reimbursed for participating and were informed about the experimental procedures prior to consenting to take part in the experiment via button click. Procedures were approved by the local Ethics Committee at the University of Regensburg.

#### Stimuli

Action stimuli corresponded to the action category labels used in Experiment 4. Specifically, category labels at the superordinate, basic and subordinate level consisted of auditory recordings of a male native German speaker reading the category labels depicted in Table 4. Visual stimuli consisted of colour photographs depicting twelve actions at the subordinate level (six exemplars per action, for a total of 72 images).

#### Design and procedure

During each trial, participants were presented with an auditory cue corresponding to a category label at the superordinate, basic or subordinate level, followed by a static image of an action that disappeared as soon as participants provided a response (for a maximum of 2 s in case no response was provided). The stimulus onset asynchrony (SOA) between the onset of the auditory cue and the onset of the image was 1100 ms. In half of the trials, the auditory cue corresponded to the action image (‘matched trials’), whereas the auditory cue and the action image belonged to different categories in the other half of the trials (‘non-matched trials’). Participants were instructed to judge whether the action depicted in the image corresponded to the auditory cue. In case of a match, participants were asked to press the key ‘f’ with the right index finger (e.g., the auditory label ‘to swim’, followed by a picture of a person swimming backstroke), and the key ‘j’ with the left index finger in case of a non-match (e.g., the auditory label ‘to ingest’ followed by a picture of a person swimming backstroke). Participants were instructed to respond as fast and accurate as possible. The experiment consisted of 216 matched trials (72 trials for each of the three taxonomic levels), and 216 non-matched trials. The order of conditions was randomized.

#### Data analysis

Data from one participant were excluded, because he did not finish the experiment. Data from two participants were excluded, because the mean accuracy was more than two standard deviations below the group mean. Data from one participant were removed, since her mean RT was more than two standard deviations above the group mean. Data from 19 participants thus were used for further analysis. Next, we calculated the mean and standard deviation of response time (RT) and accuracy, separately for the three taxonomic levels and matched/non-matched auditory cues. Mean RT was computed on the basis of correct trials. To compare mean accuracy and RT between the three taxonomic levels and matched/non-matched trials, we used a 2-factorial repeated-measures ANOVA with the factors taxonomic level (superordinate, basic, subordinate) and type of auditory cue (matched/non-matched). We used paired samples *t* test to examine pairwise comparisons, and we used Bonferroni correction to correct for multiple comparisons. Effect sizes for the results of the ANOVA are reported as partial ${\eta }^{2}$.

### Experiment 5: results

**Fig. 3:**
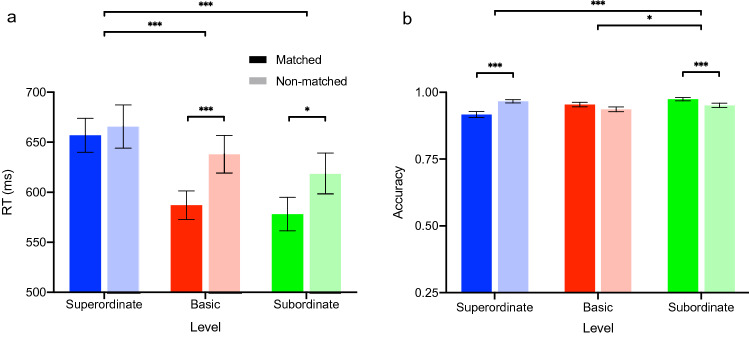
Mean RT and accuracy in the auditory priming experiment (Experiment 5). **a** Auditory cues at the basic and subordinate level led to faster responses in comparison to auditory cues at the superordinate level, and this effect was stronger if the auditory cue matched the action. **b** Auditory cues at the subordinate level led to more accurate responses in comparison to auditory cues at the basic and superordinate level. Auditory cues that matched the following action picture led to more accurate responses at the subordinate level, whereas they led to less accurate responses at the superordinate level. Error bars show SEM

Figure 3 shows the mean RT and accuracy for matched and non-matched trials as a function of the taxonomic level of the auditory cue. RT was modulated by the taxonomic level of the auditory cue [main effect taxonomic level: *F*_(2, 36)_ = 80.59, *p* < 0.001, partial ${\eta }^{2}$ = 0.82]. Specifically, in comparison to actions preceded by an auditory cue at the superordinate level [mean RT = 661.24 ms, SEM: 18.20], participants responded faster to actions preceded by an auditory cue at the basic [(mean RT = 612.49 ms, SEM: 15.63, *t*_(37)_ = 8.11, *p* < 0.001)] and subordinate level [mean RT: 598.45, SEM: 17.01, *t*_(37)_ = 11.06, *p* < 0.001]. RT did not differ between actions preceded by an auditory cue at the basic and subordinate level (*t*_(37)_ = 2.64, *p* = 0.07). Participants responded faster when the auditory cue matched the action depicted in the image [main effect for factor type of auditory cue: *F*_(1, 18)_ = 7.72, *p* = 0.01, partial ${ \eta }^{2}$ = 0.30], and this effect was modulated by the taxonomic level [interaction taxonomic level and type of auditory cue [*F*_(2, 36)_ = 9.36, *p* = 0.001, partial ${ \eta }^{2}$ = 0.34]. Specifically, in comparison to non-matched trials, participants recognized actions faster if the auditory labels matched the action, both for auditory cues at the basic (*t*_(18)_ = − 4.40, *p* < 0.001) and the subordinate (*t*_(18)_ = − 2.73, *p* = 0.01) level, but not for the superordinate level (*t*_(18)_ = − 0.65, p = 0.52).

As can be seen in Fig. 3b, accuracy was modulated by the taxonomic level of the auditory cue preceding the action image [main effect taxonomic level: *F*_(2, 36)_ = 6.97, *p* = 0.003, partial ${\eta }^{2}$ = 0.28]. Specifically, in comparison to the subordinate level (mean accuracy: 0.962, SEM: 0.006), participants responded less accurate at the superordinate (mean accuracy: 0.941, SEM: 0.07; *t*_(37)_ = − 2.45, *p* = 0.004) and the basic level (mean accuracy: 0.945, SEM: 0.07, *t*_(37)_ = 2.87, *p* = 0.03). The effect of the taxonomic level of the auditory cue was modulated by the match between the auditory cue and the action image [interaction taxonomic level × type of auditory cue: *F*_(2, 36)_ = 15.70, *p* < 0.001, partial ${\eta }^{2}$ = 0.47]. Pairwise *t* test showed that participants responded less accurate in matched in comparison to non-matched trials for auditory cues at the superordinate level (*t*_(18)_ = − 4.67, *p* < 0.001). By contrast, they responded more accurately in matched in comparison to non-matched trials for auditory cues at the subordinate level (*t*_(18)_ = 2.77, *p* = 0.01). There was no difference in accuracy between matched and non-matched trials for auditory cues at the basic level (*t*_(18)_ = 1.58, *p* = 0.13).

### Experiment 5: interim discussion

Experiment 5 showed that verbal cues at the basic and subordinate level, but not at the superordinate level, facilitate the speed of processing of observed actions, suggesting that the basic level is the most abstract level at which participants profit from an auditory cue. It is worth pointing out that these results were not fully matched by the corresponding accuracy data. Whereas participants responded more accurately in matched in comparison to non-matched trials for verbal cues at the subordinate level, in line with the RT results, this difference was not significant for verbal cues at the basic level. Moreover, unexpectedly, participants responded more accurately during non-matched in comparison to matched trials for verbal cues at the superordinate level. It is possible that for verbal cues at the subordinate level, participants profited from the fact that subordinate labels of observed actions always included the basic label action names (e.g., the subordinate level action ‘to swim breaststroke—‘Brustschwimmen’ in german includes the basic level action name ‘to swim’—‘schwimmen’ in German). Regarding the lack of a priming effect at the superordinate level, it seems likely that the superordinate action labels (locomotion, ingestion, cleaning) were simply too abstract to lead to a facilitatory effect on the processing of upcoming action images. We will return to this point in the General Discussion. We are less certain about the reasons underlying the observation that participants responded more accurately during non-matched in comparison to matched trials for verbal cues at the superordinate level.

To examine whether the three taxomomic levels also differ with respect to the speed of processing, we used a category verification task in Experiment 6 in which we systematically varied the taxonomic level of the category and the exposure duration of the action images.

### Experiment 6 (recognition): rationale

The aim of Experiment 6 was to examine whether the speed to recognize an action depends on the taxonomic level. To examine the time course of action categorization, we used a rapid category verification task in which we varied both the taxonomic level of written action labels and the exposure duration of action images (see also de la Rosa et al., 2014; Hafri et al., 2013; Mack et al., 2008). Experiments 4 and 5 revealed that the basic level includes the most common features, and that matched auditory cues speed up the processing of actions at the basic and the subordinate level, but not at the superordinate level. We thus hypothesized that participants required less time to verify the category of an action at the basic and subordinate level in comparison to the superordinate level.

### Experiment 6: methods

#### Participants

Twenty native German speakers (female: 12; age: 26 ± 5 years) joined the experiment in a behavioral lab at the Institute of Psychology at the University of Regensburg. All participants consented to take part in the experiment. They either received course credits or money as a reward for their participation. Procedures were approved by the local Ethics Committee at the University of Regensburg.

#### Stimuli

We used the same images of action stimuli as in Experiment 5 (i.e., 12 subordinate actions × 6 exemplars each, for a total of 72 images). Scrambled images were created by randomly selecting and shuffling 10 × 10 pixels squares from all action images. Written category labels (font type: Calibri) corresponded to the german action words depicted in Table 4. Stimulus presentation and data collection was implemented with A Simple Framework (ASF, Schwarzbach, 2011), which is built around the Psychophysics Psychtoolbox (Brainard, 1997).

#### Design and procedure

**Fig. 4:**
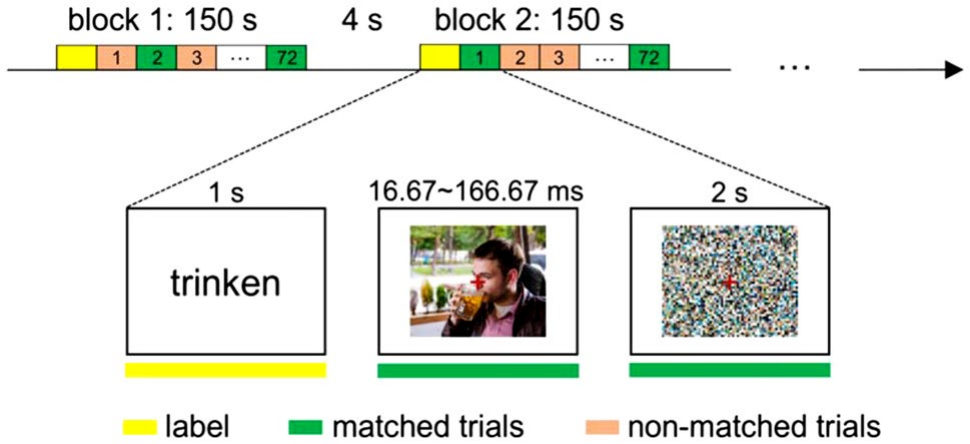
Design and procedure used in Experiment 6. Upper panel: Each block consisted of 72 trials and lasted 150 s. Lower panel: At the beginning of each block, participants were presented with a written label (in german) corresponding to an action at one of the three taxonomic levels (e.g., ‘trinken’—‘to drink’; see Table 4) for 1 s. This label was followed by a block of 72 trials. In each trial, participants were presented with an image of an action (duration: 16.67–166.67 ms in steps of 16.67 ms), followed by a scrambled mask (2 s). In each trial, participants were instructed to judge whether the action image (e.g., a picture of a person drinking a glass of beer) corresponded to the label provided at the beginning of the block (e.g., ‘trinken’—‘to drink’). In the case of a match between the action depicted in the action image and the label (‘matched trials’), participants were instructed to click the left mouse button, whereas they were asked to press the right button in the case of a non-match (‘non-matched trials’)

At the beginning of a block of trials, participants were presented with a written label of an action at one of the three taxonomic levels (e.g., “locomotion”, “to swim” or “to swim backstroke”) at the centre of the screen for 1 s, which was immediately followed by the first trial of a block (Fig. 4). Each trial consisted of an image of an action (duration: 16.67, 33.33, 50, 66.67, 83.33 or 166.67 ms), immediately followed by a scrambled mask (2 s). Exposure duration was chosen on the basis of previous studies (de la Rosa et al., 2014; Hafri, et al., 2013; Mack et al., 2008).

Participants were instructed to decide as quickly as possible whether the image just shown matched the action label. If the image (e.g., a person driving a car) matched the written label (e.g., “locomotion”), participants should click the left mouse button. If image and action label did not match, (e.g., an image of a person talking on the phone and the label “locomotion”), participants should click the right mouse button. Participants were instructed to answer after each image of an action, even if they could not recognize the action.

Each block consisted of an equal proportion of matched and non-matched trials. In total, the experiment consisted of nine blocks (three for each taxonomic level). Note that using all the category labels shown in Table 4 would have led to an imbalance regarding the number of trials at the three taxonomic levels. To avoid this, we selected three category labels at the basic level and three category labels at the subordinate level for each participant, whereas we chose all three superordinate category labels for each participant. The selected category labels were balanced across participants. Regarding the action of the selected category label, action images of all six exemplars were presented with an equal proportion within a block. That is, each combination of the selected action (six exemplars) and exposure duration (six levels) was shown exactly once within each block, for a total of 36 matched trials per block. To balance the number of matched and non-matched trials, we randomly selected the same number of non-matched trials from all possible combinations. Thus, in total, each block contained 72 trials (36 matched, 36 non-matched). The order of conditions within each block was randomized. Each block lasted approximately 2.5 min with a 4 s break between blocks. The whole experiment lasted approximately 23 min.

#### Data analysis

Data from two participants were removed, since one participant’s mean RT was more than two standard deviations above the group mean, and another participant’s mean accuracy was more than two standard deviations above the group mean. Using the data from the remaining *N* = 18 participants, we analyzed mean RT (based on correct trials) and accuracy for matched trials using a two-factorial repeated-measures ANOVA with the factors taxonomic level (superordinate, basic and subordinate level) and exposure duration of the action image (16.67, 33.33, 50, 66.67, 83.33 and 166.67 ms). Significant interactions were followed up with pairwise comparisons (Bonferroni-corrected for multiple comparisons). Effect sizes for the results of the ANOVA are reported as partial ${\eta }^{2}$.

### Experiment 6: results

**Fig. 5:**
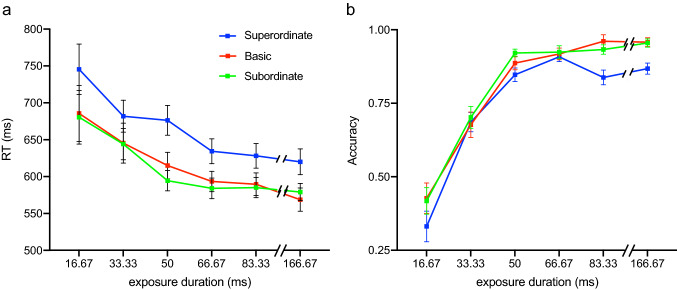
RT and accuracy for matched trials as a function of the exposure duration of the action image, separately for the three taxonomic levels. **a** Participants were faster to verify the category of actions at the basic and the subordinate level in comparison to the superordinate level across all examined exposure durations. **b** For short exposure durations, the accuracy to verify the category of actions was not affected by the taxonomic level. For long exposure durations, participants were more accurate to verify the category of actions at the basic and subordinate level in comparison to the superordinate level. At exposure duration = 50 ms, participants responded more accurately to action images preceded by category labels at the subordinate in comparison to the superordinate level

Figure 5 shows mean RT (panel A) and accuracy (panel B) during matched trials as a function of the exposure duration of the action image, separately for the three taxonomic levels. As can be seen, participants responded faster and more accurately with increasing exposure duration [main effect exposure duration: *F*_(5,80)_ = 13.51, *p* < 0.001, partial ${\eta }^{2}=0.46$]. Importantly, participants responded faster to action images preceded by category labels at the basic and subordinate level in comparison to category labels at the superordinate level [main effect taxonomic level: *F*_(2,32)_ = 9.99, *p* < 0.001, ${\mathrm{partial} \, \eta }^{2}=0.38$; pairwise comparison basic vs. superordinate level: *t*_(107)_ = 4.36, *p* < 0.001; subordinate versus superordinate level: *t*_(107)_ = 5.24, *p* < 0.001]. RT did not differ between actions preceded by category labels at the basic and subordinate level (*t*_(107)_ = − 0.35, *p* > 0.99). The effect of taxonomic level was not modulated by exposure duration [*F*_(10,160)_ = 0.51, *p* = 0.88, partial ${\eta }^{2}=0.03$].

As can be seen in Fig. 5b, accuracy was affected by taxonomic level at the longer exposure duration, but not at the shorter exposure duration [interaction taxonomic level × exposure duration: *F*_(10,170)_ = 2.00, *p* = 0.04, partial ${\eta }^{2}$ = 0.11]. At longer exposure durations (83.33 ms and 166.67 ms), participants responded more accurately to action images preceded by category labels at the basic and subordinate level in comparison to labels at the superordinate level [pairwise comparisons superordinate vs. basic at exposure duration = 83.33 ms: *t*_(17)_ = − 4.68, *p* < 0.001; at exposure duration = 166.67 ms: *t*_(17)_ = − 3.69, *p* = 0.005; superordinate vs. subordinate at exposure duration = 83.33 ms: *t*_(17)_ = − 3.84, *p* = 0.004; at exposure duration = 166.67 ms: *t*_(17)_ = − 4.08, *p* = 0.002]. In addition, at exposure duration = 50 ms, participants responded more accurately to action images preceded by category labels at the subordinate in comparison to the superordinate level [superordinate vs. subordinate at exposure duration = 50 ms: *t*_(17)_ = 5.25, *p* = 0.02]. Accuracy did not differ between actions preceded by category labels at the basic and subordinate level [*t*_(17)_ = − 1.54, *p* = 0.64], or between the superordinate and basic level [*t*_(17)_ = − 1.29, *p* = 0.43] at exposure duration = 50 ms.

### Experiment 6: interim discussion

Experiment 6 revealed that participants were faster and more accurate to verify actions at the subordinate and basic level in comparison to actions at the superordinate level. Performance did not differ between the subordinate and basic level. Whereas these results are broadly consistent with the results of Experiment 5, in particular with respect to RT, they differ from the results on the speed of object recognition reported by Rosch et al., (1976, Experiment 7), where participants were faster to verify the category of an object at the basic level in comparison to the superordinate and the subordinate level. We will return to this observation in the “General discussion”.

## General discussion

Here we aimed to investigate the characteristics of actions at different hierarchical levels. The purpose of Experiments 1–3 was to select and characterize actions at the superordinate, basic and subordinate level to be used in the following experiments. The final set of actions we selected in Experiments 1–3 for the three taxonomic levels differed with respect to their degree of abstraction (see Rating of abstraction, Supplementary Material). In Experiment 4, using a feature listing paradigm, we found that participants provided the most common features for actions at the basic level. Actions at the basic and the superordinate level were described with more distinct features than actions at the subordinate level, while actions at the subordinate level shared more information with actions from different categories at the same level than actions at the superordinate level. In Experiment 5, we found that participants are faster to respond to images of actions preceded by a matching auditory cue at the basic and subordinate level, but not for matching cues at the superordinate level. In Experiment 6, we observed that participants are faster and more accurate to verify the category of an action depicted as an image at the basic and subordinate level in comparison to the superordinate level. In sum, basic level actions were described with the largest number of common features, with more distinct features in comparison to the subordinate level, and the basic level was the most abstract level at which a verbal cue facilitated the processing of an upcoming visual action. Together, these results are in line with the view that information about action categories is maximized at the basic level. In the following sections, we are going to discuss these results in more detail in relation to previous findings.

### Comparison of taxonomic levels of objects and actions

Our feature listing paradigm (Experiment 4) revealed that participants provided more common features for actions at the basic level in comparison to the superordinate level, in line with the results reported by Rosch et al., (1976, Experiment 1). Likewise, using a similar paradigm for event categories, Rifkin (1985) and Morris and Murphy (1990) reported that participants provided more features for basic level events in comparison to events at the superordinate level, whereas they obtained no differences between the basic and the subordinate level. The results of our feature rating experiment thus suggest that, as for objects, the basic level contains more inclusive information about actions than the superordinate level, and that information at the basic level is best suited to determine similar items within a category and distinctions between other categories. Moreover, the larger number of distinct features at the superordinate level in comparison to the subordinate level suggests a higher distinctiveness between actions at this level. By contrast, the higher number of shared features at the subordinate level suggests that actions at this level are more cohesive and thus less distinguishable (see also Rosch, 1978).

The results of our auditory priming paradigm (Experiment 5), with faster responses in matching in comparison to non-matching trials for the basic and subordinate level, and the absence of a difference between matching and non-matching trials for the superordinate level, are in line with the results of the priming experiment reported by Rosch et al. (1976) for objects.

Our category verification task (Experiment 6) revealed that participants recognized actions faster at the basic level in comparison to the superordinate level, while we obtained similar results for the basic and the subordinate level, in line with the results of Experiment 5. Note that the absence of a difference between the basic and the subordinate level we obtained both in Experiments 5 and 6 is in line with the results of the feature-listing paradigm used by Rifkin (1985) and Morris and Murphy (1990) for events. Likewise, Rosch et al. (1976) reported faster responses in matched in comparison to non-matched trials for primes at the basic and the subordinate level, but no difference between matched and non-matched trials for primes at the superordinate level in a priming task. The priming effect did not differ between the basic and the subordinate level. By contrast, Rosch et al. (1976) obtained faster responses at the basic level in comparison to the superordinate and the subordinate level in an object recognition task (Experiment 7 in their study).

In summary, the difference between the basic and the superordinate level has been reported consistently across paradigms and stimulus domains (objects, actions). By contrast, the difference between the basic and the subordinate level is less consistent, with some studies reporting a difference, while other studies obtained no such difference. Finally, de la Rosa et al. (2014) directly compared the average recognition time for objects (e.g., car) and social interactions (e.g., to hug) at the basic and the subordinate level as a function of exposure duration (similar to the paradigm used in Experiment 5 in the current study). They found that both objects and social interactions were recognized faster and more accurately at the basic than at the subordinate level. However, this difference was substantially larger for objects than for social interactions.

What might determine under which circumstances categories at the basic level and the subordinate level are processed in a similar or in a different way? First, prior studies found no difference between the basic and the subordinate level in terms of speed and accuracy for the recognition of objects in experts from the corresponding object fields (Johnson & Mervis, 1997; Tanaka, 2001; Tanaka & Taylor, 1991). This raises the possibility that some of the differences between the subordinate and the basic level obtained in previous studies might be due to differences in terms of familiarity or expertise with the objects at the two taxonomic levels, whereas participants were likely to be highly familiar with the actions at the basic and subordinate level in the current study. Second, a possible reason for the lack of a difference in terms of priming effects (Experiment 5) and the speed of recognition (Experiment 6) between actions at the basic and the subordinate level observed in the current study lies in the fact that labels of actions at the subordinate level (e.g., ‘to swim breaststroke’—‘Brustschwimmen’) always included the label of the basic level (e.g., ‘to swim’—‘Schwimmen’). Note that the same holds for some of the object categories examined by Rosch et al., (1976; e.g., ‘desk lamp’—subordinate level/‘lamp’—basic level), but not for all of them (e.g., ‘Levis’—subordinate level/‘pants’—basic level). Third, and not mutually exclusive with respect to the previous two points, participants in the current study may have profited from the presence of objects in the majority of the subordinate action names, which might have abolished any differences between the basic and the subordinate level. Future studies are required to examine the exact circumstances under which the number of common features obtained in feature listing paradigms as well as the speed and accuracy for the recognition of objects and actions differs between the basic and the subordinate level.

In sum, while we noticed some differences with respect to previous studies, most of our results are in line with the results previously reported for the basic level advantage of objects and events. Together, the results of Experiments 4–6 suggest that actions at the basic level had maximum cue validity. Moreover, they reflect the effect of cognitive economy, with a trade-off between distinctiveness and informativeness.

### The role of stimulus format

In comparison to visual representations, verbal descriptions are more specific and informative regarding the taxonomic level of a category (Morris & Murphy, 1990; Rosch et al., 1976). As an example, we can refer to the subordinate (‘to eat an apple’), basic (‘to eat’) and superordinate level (‘to ingest’) verbally, whereas it is difficult to depict the three different levels in the visual format (unless the visual format is embedded in a task, such as a category verification task or a priming paradigm). Another important difference between the verbal and the visual format lies in the fact that a number of studies on actions in the visual domain emphasized the way in which these actions are performed and the goal that one aims to achieve with these actions (e.g., Spunt et al., 2016; Hamilton & Grafton, 2006; Wurm & Lingnau, 2015). Consequently, it has been proposed that one important principle underlying the representation of observed actions is an organization according to their goals (Hamilton & Grafton, 2006; Tunik et al., 2005).

Regarding verbal material, Schank and Abelson (1977) described the internal structure of scripts, such as ‘going to a restaurant’, with a specific emphasis on action primitives, such as move, speak, or ingest. However, as mentioned also by Morris and Murphy (1990), this line of research was concerned about the relationship between parts of scripts (such as ‘waiting to be seated’, ‘ordering food’) rather than the relationship between different semantic categories (such as ‘going to a restaurant’ and ‘visiting a museum’). By contrast, other studies on actions in the verbal format focused on the role of the grammatical class (in particular, verbs vs. nouns; see, e.g., Peelen et al., 2012), the distinction between action versus non-action verbs (e.g., Papeo & Lingnau, 2015; Papeo et al., 2015), and semantic categories or semantic fields (e.g., Pinker, 1989; Talmy, 1985). Regarding the latter, a number of studies focused on the (horizontal) organization of semantic categories, such as change of location, communication and change of state (e.g., Vinson & Vigliocco, 2008), corresponding to the superordinate level used in the current study.

### The role of different types of features

In their experiment 2, Rosch et al. (1976) instructed participants to list motor (i.e., body and muscle) movements associated with specific objects. They found that participants provided fewer motor movements for objects at the superordinate level in comparison to objects at the basic and subordinate level. By contrast, they observed no difference for motor movements associated with objects at the basic and subordinate level. These results illustrated that the basic level of objects was the most inclusive level at which many motor movements interacted with objects. Experiment 2 by Rosch et al. (1976) provided a good foundation for investigating motor features of objects across taxonomic levels. In line with this view, a number of studies highlighted the importance of motor- and body-related features, such as movement kinematics (Cavallo, et al., 2016), movement force (Casiraghi et al., 2019) and the amount of arm movement and hand posture (Watson & Buxbaum, 2014) for the processing of observed actions (see also de Gelder & Poyo Solanas, 2021, for a recent discussion of the importance of midlevel features).

Inspired by these previous studies, we further subdivided the common features into movement, body-part and object features in an exploratory analysis. Features related to body parts and objects did not differ between taxonomic levels. By contrast, participants used more movement features to describe actions at the basic level in comparison to the superordinate level, in line with the idea that the basic level is most inclusive also with respect to movement-related information (see also Rosch et al., 1976, Experiment 2). However, features related to motor movements are not the only features that play a role in the categoriziation of actions. As an example, several recent studies emphasized the role of high level features, such as the target (e.g., a person or an object; Tarhan & Konkle, 2020; Wurm et al., 2017) or the emotional valence of an action (e.g., Kroczek, et al., 2021; Portugal et al., 2020).

### Future directions

The current set of experiments lays the foundation for a number of interesting lines for future research. As an example, under which conditions is there a behavioral advantage for actions at the basic in comparison to the subordinate level, and under which circumstances do the two levels lead to similar behavioral effects? To which degree are the behavioral effects modulated by the typicality of an observed action (see also Murphy & Brownell, 1985, for typicality constraints on the basic object advantage), or by the presence or absence of a concrete target object? Moreover, it will be interesting to examine which taxonomic level is learned first by children, and whether there are differences in the processing of obseved actions at the different taxonomic levels for young and elderly adults. Finally, it will be important to establish a link between the hierchical organization of actions examined in the current study and the underlying neural representation in space and time.

## Conclusions

Understanding whether a hierarchical structure may be an emergent property underlying the organization of observed actions is key to answering the broader question about how the human brain extracts and organizes information from the surrounding world in a flexible way. The current study extends previous studies focusing on the horizontal organization of observed actions by examining the vertical organization of actions (see also Vallacher & Wegner, 1985; Rifkin, 1985; Morris & Murphy, 1990). Our findings are in line with the view that there is a basic level advantage not only for objects, but also for actions.

## Supplementary Information

Below is the link to the electronic supplementary material.

Supplementary file1 (DOCX 34 kb)
