# Supplementary material for: The characterization of actions at the superordinate, basic and subordinate level
Source: Psychol Res. 2021 Dec 14;86(6):1871–91. doi: 10.1007/s00426-021-01624-0 (PMC9363348; doi:10.1007/s00426-021-01624-0)
Supplement: Supplementary file 1 — Supplementary file1 (DOCX 34 kb) [file 426_2021_1624_MOESM1_ESM.docx]

**Supplementary Material**

**Experiment 1: Rating of semantic similarity**

**Table S1.** Basic level action verbs in their infinitive forms (and their German translation) used in Experiment 1 (right column), together with the corresponding superordinate category labels (left column).

| **Category labels (superordinate level)** | **Action verbs (basic level)** |
| --- | --- |
| communication (*Kommunikation*) | to read (*lesen*) |
|  | to write (*schreiben*) |
|  | to tell (*erzählen*) |
|  | to sing (*singen*) |
|  | to talk (*unterhalten*) |
|  | to shout (*schreien*) |
|  | to call (*rufen/ anrufen*) |
| locomotion (*Bewegung*) | to climb (*klettern*) |
|  | to run (*laufen*) |
|  | to swim (*schwimmen*) |
|  | to drive (*fahren*) |
|  | to walk (*gehen*) |
|  | to jump (*springen*) |
|  | to dance (*tanzen*) |
| to ingest (*Nahrungsaufnahme*) | to eat (*essen*) |
|  | to drink (*trinken*) |
|  | to feed (*füttern*) |
| change of state (*Veränderung des Zustandes*) | to cook (*kochen*) |
|  | to brush (*putzen*) |
|  | to break (*brechen*) |
| to learn (*Lernen*) | to teach (*lernen*) |
|  | to learn (*lesen*) |
|  | to study (*studieren*) |
|  | to memorize (*merken*) |
| grooming & body care (*Pflege & Hygiene*) | to wash (*waschen*) |
|  | to wear (*anziehen*) |
| perception (*Wahrnehmung*) | to observe (*beobachten*) |
|  | to feel (*fühlen*) |
|  | to touch (*anfassen*) |
|  | to see (*ansehen*) |
|  | to hear (*hören*) |
| creation & transformation (*Erzeugung & Herstellung*) | to build (*bauen*) |
|  | to sew (*nähen*) |
|  | to paint (*malen*) |
|  | to draw (*zeichnen*) |

**Instructions**

Scale: 1= completely dissimilar, 4=moderate, 7= completely similar

Below are pairs of action phrases. Please rate how dissimilar versus similar the meaning of each pair of phrases is on a scale from 1 to 7. E.g., the meaning of the words ‘taking a shower - jumping’ can be considered completely dissimilar in meaning, so we could rate their similarity as 1. The meaning of the words ‘taking a shower – taking a bath’ is very similar in meaning, so we could rate it as 7. Please consider only the meaning of these two phrases.

**Fig. S1.** Silhouette index. The silhouette index provides an estimate of the averaged distance between clusters as a function of the number of clusters. In a range of 2 to 10 clusters, we obtained the highest silhouette index for six clusters.

**Experiment 2: Taxonomic depth task**

**Instructions**

*First part:*

In this part, you will see a series of single phrase presented at the top of the screen. Please type down which kind of superordinate category this phrase belongs to. E.g. when ‘watching TV' shows up at the top of the screen, you could type down ‘relaxation-related action’. You have 30 s to type down the phrase. Please do your best in this study, your performance is important to our study.

*Second part:*

In this part, you will see a series of single words presented at the top of the screen. Please type down which types of subordinate categories belong to this word. For example, when the ‘relaxation-related action’ shows up at the top of the screen, you could type down ‘listening to music’, ’painting’… You have 30s to type your answer down. Please focus on this study, your performance is important to our study.

**Experiment 3: Ratings of Relatedness, Complexity, and Abstraction**

**Instructions**

**Relationship (weak, strong)**

In the following you will be provided with a generic term (e.g. ‘Sport’, ‘Celebration’) describing an action at the top of each page. Underneath, you will be provided with several action words. Please read each action word and judge the relationship with the generic term presented at the top of the page. For example, if you could judge the relationship between *watching TV* and *Sport* to be very weak, you could rate it as 1. By contrast, if you judge the relationship between *playing a board game* and *celebration* as very strong, so you could rate it as 7.

Scale: 1 = weak relationship, 4 = neutral, 7= strong relationship

**Complexity (simple/ complex)**

In the following, you will be provided with a number of brief German phrases. Please read each phrase and judge how easy or complex it is to perform the described action. For example, *making a fist* is very easy to do, so you should assign a 1 (easy) to this action. In contrast, *playing the violin* is a complex action, which is why this action should be assigned a 7 (complex). Please consider all levels of the scale when making your assessment.

**Abstraction (concrete/ abstract)**

In the following you are provided with a number of phrases describing human actions. Your task is to rate the level of abstraction of each phrase on a scale from 1 to 7. To make sure you understand what we mean by “concrete” and "abstract", please keep the following dictionary definitions in mind when making your ratings:

• Concrete: existing in a material or physical form; real or solid; not abstract

• Abstract: existing in thought or as an idea but not having a physical or concrete existence

For example, *peeling a potato* is very concrete, so you could rate the level of abstraction as 1; *buying a house* is very abstract, so you could rate it as 7.

If you think that a phrase is completely concrete, rate it as 1. If instead you think it is completely abstract, rate it as 7. If you think it is just as concrete as it is abstract, rate it as a 4. The other numbers represent intermediate points on the scale. Please use the entire range of the scale when making your ratings.

**Experiment 4: Feature Listing Paradigm**

**Instructions**

In this experiment you will be presented with phrases describing different actions (e.g. "to write an SMS") at the top of the page. Your task is to write down features of this action. Such features could be body parts involved in the action, the target of the action, the type of movements involved, specific postures, the duration, the force required, the speed etc. Of course, not all of these features will always be relevant to a particular action.

Please type down as many features as possible in two minutes. Here are some examples:

*to write*

*Action features:*

hold the pen between thumb and index finger

move hand

swinging movement from left to right

pen

paper

communication

small, fast movements

precision

*To write an SMS*

*Action features:*

type

mobile phone

write

communication

message

use thumb and index finger

contact someone

fast movements

*Communication*

*Action features:*

targeting another person

exchange of knowledge

mouth

emotional expression

eye contact

body language

speak

letter

**Experiment 4: Rating of abstraction**

**Participants:** Twenty native or fluent German speakers (female: 14, age: 27± 4 years old) took part in this experiment. All participants consented to participate in the study via button click, after they were informed about the instruction of the questionnaire.

***Stimuli.*** We used the german action category labels at the three taxonomic levels as shown in Table 4.

***Procedure.*** The questionnaire was conducted with an online platform ([www.soscisurvey.com](http://www.soscisurvey.com)). After being provided with a written instruction (see details below), participants were provided with each of the action labels in a randomized order, one after the other. Participants were asked to judge the abstractness of action labels on the scale of 1 to 7 (1: very concrete, 7: very abstract).

***Data analysis.*** We used the Kruskal-Wallis H test to compare ratings of abstraction between the three taxonomic levels. We used Dunn’s post hoc test and corrected multiple tests using Bonferroni correction. All statistical analysis was implemented in SPSS.

***Results:*** The mean rank of abstractedness differed between taxonomic levels (mean rating superordinate level: 5.10, basic level: 4.03, subordinate level: 1.45; H_(2)_ = 288.75, p < .001; effect size: $\eta^{2}$ = 0.69). Dunn’s post-hoc test with multiple Bonferroni multiple corrections showed that participants regarded action category labels at the superordinate level as the highest abstract level, while they rated action category labels at the basic level as the intermediate level and action category labels at the subordinate level as the most concrete level [superordinate vs subordinate: p < .001; superordinate vs basic: p < .05; basic vs subordinate level: p < .001].

***Instruction for rating of abstraction:*** In the following, you will be provided with a number of phrases describing an action (e.g., to walk; to eat an apple). Your task is to indicate on a scale from 1 to 7 how abstract these actions are (1: very concrete; e.g., 'to peel potatoes'; 7: very abstract, e.g., 'to communicate'). If a term is equally abstract and concrete, please assign it a 4 (equally abstract and concrete). All other digits on the scale fill in the spaces meaningfully. Please consider all levels of the scale when making your assessment.

You may refer to the following definitions:

- Concrete: existing in a material or physical form; real or solid; not abstract

- Abstract: existing in thought or as an idea but not having a physical or concrete existence

1=perfectly concrete;

4=equally abstract and concrete;

7=perfectly abstract
